# Interpersonal counselling for adolescent depression delivered by youth mental health workers without core professional training: a feasibility randomised controlled trial study protocol

**DOI:** 10.1186/s40814-020-00733-8

**Published:** 2020-12-10

**Authors:** Gabriel Abotsie, Viktoria Cestaro, Brioney Gee, Jamie Murdoch, Thando Katangwe, Richard Meiser-Stedman, Lee Shepstone, David Turner, Susie Tulk, Sharon Jarrett, Jon Wilson, Tim Clarke, Bonnie Teague, Paul Wilkinson

**Affiliations:** 1 Norfolk and Suffolk NHS Foundation Trust, Norfolk, UK; 2 Norwich Medical School, University of East Anglia, Norwich, UK; 3 School of Health Sciences, University of East Anglia, Norwich, UK; 4 Department of Clinical Psychology, University of East Anglia, Norwich, UK; 5 Suffolk County Council, Suffolk, UK; 6 Research and Development, Norfolk and Suffolk NHS Foundation Trust, Norfolk, UK; 7 Department of Psychiatry, University of Cambridge, Cambridge, UK; 8 Children and Young People’s Services, Cambridgeshire and Peterborough NHS Foundation Trust, Cambridge, UK

**Keywords:** Depression, Interpersonal Counselling, Interpersonal Counselling for Adolescents, Young people, Adolescents, Early Help, Youth Workers, Local Authority

## Abstract

**Background:**

Depression is a common health problem during adolescence and is associated with adverse academic, social and health outcomes. To meet the demand for treatment for adolescent depression, there is a need for evidence-based interventions suitable for delivery outside of specialist Child and Adolescent Mental Health Services (CAMHS). Interpersonal Counselling for Adolescents (IPC-A) is a brief manualised intervention for adolescent depression suitable for delivery by staff who are not qualified health professionals following participation in a brief training course. While initial piloting within Local Authority services has generated promising results, the effectiveness and cost-effectiveness of IPC-A has yet to be established. This study aims to assess the feasibility of a randomised controlled trial (RCT), evaluating the effectiveness and cost-effectiveness of IPC-A delivered by staff without core professional training in comparison to current provision.

**Method:**

Feasibility RCT with process evaluation using ethnographic methodology. Eligible young people (*n* = 60) will be randomised in a 1:1 ratio to receive either IPC-A or treatment as usual (TAU). Participants will be assessed pre-randomisation (baseline) and followed up at 5, 10 and 23 weeks post-randomisation. A parallel process evaluation will generate understanding of intervention implementation across services and explore the acceptability of the intervention from the perspective of young people and other key stakeholders.

**Participants:**

Young people aged 12–18 years presenting to non-specialist services with symptoms of depression. Youth workers, young people and stakeholders will participate in the process evaluation.

**Discussion:**

The need for effective and accessible interventions for young people with mild/sub-threshold depression who, in most cases, do not meet the threshold for mainstream mental health services is long overdue. The primary output of this feasibility trial will be the design of the subsequent full-scale trial. If the results of the current study indicate that this would be feasible, we intend to progress to a multi-site, assessor-blind, superiority RCT of the effectiveness and cost-effectiveness of IPC-A in comparison to TAU for adolescents presenting to non-specialist services with depressive symptoms. If satisfactory solutions to any problems encountered cannot be identified, alternative research designs will be considered. If proven effective, an IPC-A training programme could be implemented.

**Trial registry:**

ISRCTN registry, ISRCTN82180413, Registered 31 December 2019.

## Background

Depression is a common health problem during adolescence. Adolescent lifetime prevalence of major depressive disorder (MDD) is 11–20% [1, 2]. However, mild/sub-threshold depression is much more common in adolescents than full MDD [3] (People with mild/sub-threshold depression show some clinical symptoms of MDD but number of symptoms is just below or only just above the diagnostic threshold.) Such mild depression is associated with significant personal and public health consequences [4] and is a strong predictor for future onset of full [5]. Sub-threshold depression has a lifetime prevalence rate of 26% [6] and about 30% of affective disorders that occur in adulthood start in adolescence [7]. Depression in adolescence predicts a range of adverse outcomes in adulthood, including ongoing mental health problems [8], poorer physical health [9], and social, legal and financial problems [10], and is the most prevalent psychiatric disorder in young people who die by suicide [11]. The total annual cost of depression in England has been estimated to be at least £20.2 billion [12]. However, there is evidence that early and timely psychological intervention can prevent relapse and recurrence [13], and therefore, intervening early, before depression symptoms become severe, could generate substantial savings.

In the UK, the majority of mental health services for young people are commissioned through a tiered system; tier 1 represents early intervention and prevention services, provided by schools, school nurses and General Practitioners; tier 2 involves early help and targeted services and may be provided by young people’s teams, youth support services, family support teams, education support centres and education psychologists; tier 3 consists of specialist Child and Adolescent Mental Health Services (CAMHS); and tier 4 is inpatient mental health services. Most adolescents seeking treatment for depression have mild disorder [14]. In the UK, such cases of mild depression are not likely to meet treatment thresholds for specialist tier 3 services. Instead, young people with mild depression are seen by staff working in local authority child and family services or tier 2 NHS-funded mental health services often delivered by third-sector or voluntary agencies. Most of those working with depressed young people within these non-specialist services do not have core mental health professional training, e.g. psychiatrist, psychologist, registered mental health nurse and psychological therapist (e.g. CBT therapist, family therapist) and have no formal training in delivering evidence-based treatments for people with depression.

Current guidelines for the treatment of mild depression in children and young people [15] recommend simple non-specific psychosocial strategies, such as non-directive supportive therapy (which mainly involves active listening rather than developing specific strategies to improve symptoms). A recent large network meta-analysis has shown that while non-directive supportive therapy is better than a waiting list (i.e. no treatment) for adolescent depression, it is not significantly better than psychological placebo [16]. It is important to note that the primary studies included in this meta-analysis took place in a range of services for a range of severities of depression. No randomised controlled trials have taken place in the tier 1/2 services described above, where most cases of mild depression are treated in the UK. Thus, there is a clear lack of evidence as to how to treat young people in these services [17–19].

Interpersonal psychotherapy (IPT) is a NICE-recommended first-line treatment for adolescents with moderate to severe depression. IPT helps patients to understand the two-way links between their depressive symptoms and current interpersonal relationships. It also helps patients to improve their interpersonal relationships. In doing so, it aims to reduce depressive symptoms. Whereas non-directive supportive therapy aims ‘to help patients accommodate to existing reality rather than try to help them change it’ [20], IPT focuses on helping patients to take active steps to improve their relationships in order to decrease their depressive symptoms. Theoretical influences on IPT include Adolf Meyer’s ‘psychobiological’ approach, which emphasized patients’ current interpersonal and psychosocial experiences [21] and Harry Stack Sullivan’s ‘interpersonal’ approach, which conceptualised psychiatry as the scientific study of people and interpersonal processes [22].

Meta-analyses have demonstrated IPT to be superior to control treatments for depression in both adults [23] and adolescents [16] and to lead to similar outcomes as cognitive-behaviour therapy in both age groups. Crucially, IPT has been shown to be significantly more effective than supportive counselling for depressed adolescents [24]. Given the importance of interpersonal relationship difficulties in the causation of adolescent depression [17], and the developmental priority given to interpersonal relationships during adolescence, this approach has high face validity for this age group.

However, in common with other evidence-based treatments for adolescent depression, IPT must be delivered by those with core mental health professional training. As such, it is unlikely to be a feasible treatment option outside of specialist CAMHS in the UK. Interpersonal counselling (IPC) is an adaptation of IPT with three main differences: the treatment duration is shorter (3–6 sessions), it is designed for clients with mild depression, and it can be delivered by non-mental health professionals after participation in a brief (2-day) training course. If found to be an effective treatment, training existing workers in non-specialist young persons services as IPC therapists could facilitate a rapid and relatively low-cost expansion of the therapy workforce in line with NHS England and government policy.

IPC has been found to be an effective treatment for adults with mild to moderate depression. A pilot study [25] which compared IPC and IPT concluded that IPC was equally an effective intervention for treating mild to moderate depression in primary care setting. In a randomised control trial conducted in primary care [26], IPC was compared with selective serotonin reuptake inhibitor (SSRI) amongst patients with major depression, and it was found that IPC achieved significantly higher rates of remission in adults with mild depression. An adapted form of IPC designed to meet the needs of young people (IPC-A) has recently been developed and piloted by members of the research team of this study (PW and VC): six local authority family support workers in the UK were trained in IPC-A [27]. Evaluation of the single-arm pilot demonstrated that young people undergoing IPC-A experienced an improvement in depressive symptoms and that the therapy was acceptable to young people and therapists. However, it is unknown whether IPC-A is more effective than the current treatment as usual (TAU) for young people presenting with depressive symptoms in these services. A randomised controlled trial of IPC-A vs TAU is needed to answer this question, minimising bias and confounding. However, large-scale RCTs have not been conducted in this staff/participant population, and so, it is unsure whether such an RCT would be practical. Hence, a feasibility RCT is needed first.

This study is intended to provide the information needed to progress to a full-scale randomised controlled trial of IPC-A delivered by staff without core professional training (referred to in this paper as ‘youth mental health workers’).

## Aims and objectives

The ICALM study will inform a future trial of the effectiveness and cost-effectiveness of the intervention (IPC-A). The aim of the proposed research is to answer the following feasibility questions which arise from the variability in service models across providers of non-specialist mental health support for young people:

1. Are trial procedures, including recruitment (of participants and therapists), randomisation, research assessments and follow-up, feasible and acceptable to support the conduct of a future RCT?
2. How are IPC-A and current treatment as usual (TAU) delivered and how and why does intervention delivery vary across differing service contexts?
3. To what extent does contamination of the control arm occur and should it be mitigated against in a future trial?
4. Does the interval estimate of benefit of IPC-A over TAU in depression scores at post-treatment include a clinically significant effect?

## Methods

### Design

The study will answer the research question: Is a full-scale RCT of interpersonal counselling for young people with mild depression delivered in non-specialist community services feasible? In this feasibility RCT, 60 eligible young people will be randomised on a 1:1 ratio to receive to IPC-A or treatment as usual. Participants will be assessed at baseline (pre-randomisation) and followed up at 10 weeks and 23 weeks. The feasibility trial will recruit young people presenting with low mood, who are receiving support from participating services in Norfolk and Suffolk Counties in the UK. A process evaluation has been incorporated to explore how the intervention is implemented across the two sites. Qualitative data will be collected by site profile questionnaires, observations of IPC-A training workshops and supervision, video/audio recordings of treatment sessions (both IPC-A and TAU), interviews with participants (and parents) from the IPC-A and TAU arms and focus groups with youth mental health workers and wider stakeholders.

### Participants

#### Inclusion and exclusion criteria

Participants will be young people accessing participating services via each service’s standard referral pathways as detailed below. Young people will be triaged and assessed according to each service’s standard procedures. If this assessment identifies low mood as a presenting difficulty, the case will be discussed with a clinical member of the research team (without identifying the young person) to ascertain likely suitability for the trial. The service will have the option of using the RCADS depression scale to help determine suitability, with a cut-off of 11 or over suggesting suitability (this cut-off will not be an absolute). RCADS is a primary outcome measure for the feasibility trial, and for that matter, it will be completed as part of the baseline assessment irrespective of its use by services to determine eligibility. In line with the approach used successfully in the pilot, study eligibility criteria have been kept to a minimum to reflect service eligibility criteria in order to increase the external validity of the trial in the context of non-specialist services.

##### Inclusion criteria

- Aged 12–18 years
- Seeking help for low mood (as the primary presenting difficulty)
- Of a level of illness where they would normally receive treatment from the participating service
- Able to provide written informed consent or, for under 16 years, written informed assent and parent/guardian consent

##### Exclusion criteria

- Learning disability necessitating non-mainstream schooling
- Current psychotic disorder
- Current substance dependence
- Current significant suicidal ideation (K-SADS-PL—‘suicidal ideation’ threshold—‘often thinks of suicide and has thought of a specific method’)

There will not be a numerical upper severity threshold. The upper threshold comes under ‘Of a level of illness where they would normally receive treatment from the service’. An interesting outcome of our initial IPC single-arm pilot was that some young people with severe depression (according to ratings questionnaires) are routinely treated by some early help services. Reasons are multiple. It is important to examine this in the wider range of services in the planned study; indeed, the planned study will more formally identify the range of depression severity seen in these services and what treatments are effective here. But the purpose of this study is not to examine/change referral thresholds, rather to investigate optimal treatments for young people in this service.

### Setting

The trial will be conducted in two counties in England. While the sites are in the area served by one NHS mental health trust, Tier 1/2 services or services for mild depression are not delivered by this mental health trust, as the severity of illness of young people is generally below the thresholds for NHS specialist child and adolescent mental health services. Treatment at this level is delivered by a range of services locally.

Within the two counties, these non-specialist mental health support services for children and young people are provided by county councils such as Early Help teams, Young People’s teams, Family Support services, school nurses, and NEET (not in education, employment or training) teams. In addition, two publicly commissioned independent counselling organisations will also be involved in the study. Such tier 1 or 2 services provide early interventions to children and young people with mild mental health problems and/or difficult family circumstances such as parental drug and alcohol dependency, poor mental health and domestic abuse. Practitioners working in these services are not qualified mental health practitioners but may have some training in counselling, family work and social care. Staff delivering the IPC and TAU interventions will be employees of these organisations, doing this as part of their regular job. Having this variety of services involved will give a good balance of generalisability while making the study feasible within the cost envelope.

### Intervention: Interpersonal Counselling for Adolescents (IPC-A)

IPC-A is a brief manualised psychological intervention, derived from IPT. IPC helps clients to identify the reciprocal interaction between their current depressive symptoms and interpersonal relationships, with a focus on one of four domains: grief, relationship disputes, big changes, and loneliness and isolation. The therapist works with the client to identify effective strategies to deal with their interpersonal problems, which should improve depressive symptoms.

IPC-A is an adapted form of IPC designed to suit the needs of adolescents. The intervention is delivered over three to six (30–60 min) sessions, depending on participant needs. IPC-A is based on the manual developed by Weissman et al. [28]. The core interventions are the same as in the adult manual; however, language and examples are adapted to make them more relevant to adolescents. While we are testing efficacy of IPC-A, the manual is available for researchers interested in conducting research on it, on request from PW, the senior author of this paper. IPC-A arm participants will also have access to standard health and care provision (treatment as usual) throughout their participation, such as family work; the extent to which provision of IPC-A alters use of these services will be monitored using the Client Service Receipt Inventory (CSRI).

Staff to be trained as IPC-A therapists will receive two full days of initial training, followed by weekly supervision until adequate competency levels have been demonstrated (two sessions for each of two cases above quality threshold on IPC audiorecording rating scale [27]). Detailed training on complicated issues such as grief, and weekly supervision is likely to reduce harm from therapy (which will itself be asked about in the process evaluation). Following successful completion of this training, therapists will receive monthly clinical supervision. Where possible, supervision will be provided in a group format to allow therapists to explore the theory and practice of IPC through engaging in shared discussion of real-world cases. Each supervision session will last up to 1.5 h. Supervision will be delivered by PW, VC and ST, all trained IPT/IPC supervisors, with VC having overall responsibility for coordinating the provision of clinical supervision. Attendance at and costs of training and supervision will be recorded as therapy costs.

### Control: treatment as usual

The control arm will receive ‘treatment as usual’ (TAU): the standard support provided by services. Participants will not be denied access to any treatment option available as part of current provision; however, staff providing individual support to TAU participants will not have attended any IPC-A training and will not receive any IPC-A supervision, to minimise contamination. Staff trained as IPC-A therapists will be required by contract not to discuss any aspect of their training or supervision with colleagues not trained in IPC-A.

There appear some variations in TAU options offered to young people by participating teams. County differences in service commissioning, locality or team differences in service provision may contribute to these variations. The process evaluation arm of this study and the CSRI has been designed to capture the interventions that constitute TAU as part of the study. At its core, all TAU encompasses a risk assessment framework used together with the families to identify and assess risk and appropriately plan to meet the needs of young people and their family. Where appropriate, practitioners offer counselling, themed group sessions, advice and information for parents/carers, and telephone support. Early Help Family Practitioners offer direct work to children and young people and their families who may focus on building self-esteem, supporting access to other services, supporting reintegration into education (if applicable) and working with the young person and families to understand and prevent risk.

Although the practitioners delivering these services in both IPC and control arms do not have core mental health professional training, they may consult with or offer a joint appointment with a mental health professional (e.g. primary mental health worker or clinical psychologist) or signpost/refer the young person to other local services.

### Recruitment procedure

Young people will be triaged and assessed by the referring service according to each service’s standard procedures. If this assessment identifies low mood as a presenting difficulty, the case will be discussed with a clinical member of the research team (without identifying the young person) to ascertain likely suitability for the trial. Potentially suitable young people (and/or parents/carers if under 16) will be invited to participate. If they express an interest, consent will be given to the service to pass on their details to the research team. We are requesting teams only pass potential cases to the trial if therapy in both IPC and TAU can start within a couple of weeks of the baseline assessment, so differential waiting times do not bias results.

Those who express an interest will be contacted by the trial’s research practitioner who will further explain the study, answer any queries and provide copies of participant information sheets. Potential participants will be given at least 48 h to read and consider the information before being asked for consent.

If the young person wishes to participate following this process, the research practitioner will arrange a meeting at a convenient venue (e.g. their home address, school/college or a community venue), where the young person will be asked to complete a consent form (if 16 or over) or assent form (if under 16) at the start of the first research assessment to document the informed consent/assent process and their willingness to participate. For young people under 16, in addition to the child’s assent to participation, the consent of a parent or carer (adult with parental responsibility) will be required for the young person to be included in the study. Consent to participate in an interview as part of the process evaluation will be sought during the main consent procedures. However, it will not be a requirement that a young person/parent consents to a process evaluation interview in order to be included in the study.

After informed consent has been appropriately obtained, participants will be asked to complete all baseline assessment measures. Only those who meet the eligibility criteria outlined above will be randomised. It is envisaged that a close liaison between the research practitioner and referring teams, and the subsequent telephone conversation with potential participants before baseline visit will ensure most referrals will conform to the eligibility criteria before the baseline assessment. However, in cases (which will be rare) where young persons do not meet the eligibility criteria after baseline assessment, it will be conveyed to them sensitively, and the research practitioner will liaise with the referring team to ensure the appropriate service is sought for the young person.

**Fig. 1:**
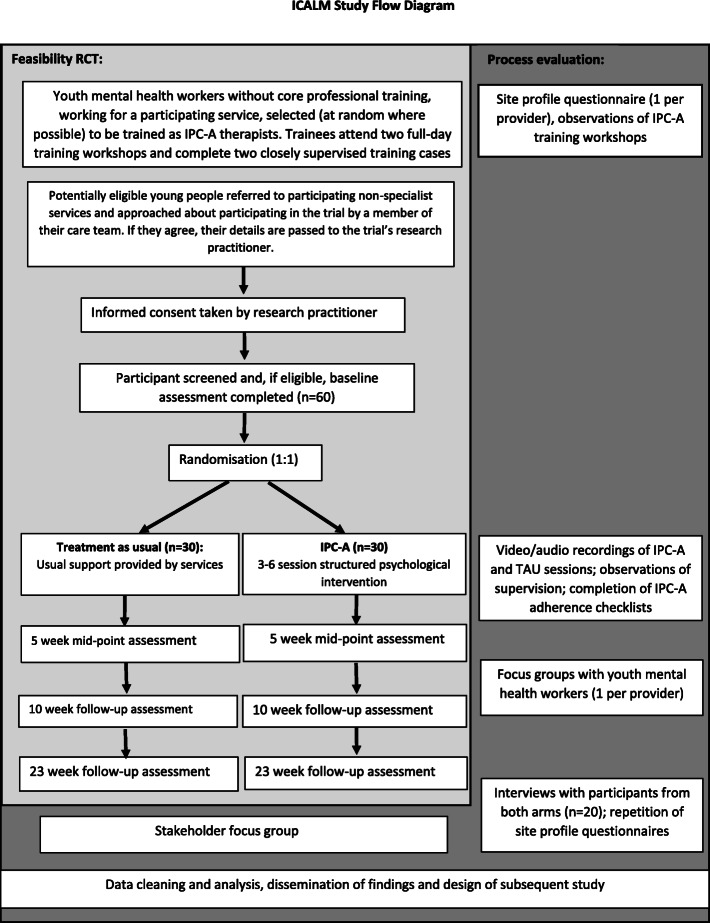
Flow chart

All staff members to be trained in the intervention will be given a verbal explanation of the objectives of the study, what he or she will be asked to do if they choose to participate and the possible risks and benefits of participation. Participation in the trial is not a condition of training. Participation in the process evaluation (listening to audiotapes of therapy and/or focus groups) is optional; staff will be given participant information sheets and asked for informed written consent to take part. Figure 1 is a flowchart diagram for this study.

### Sample size

Sixty eligible young people will be randomised all together. The sample size is not based upon estimation of efficacy but is in keeping with published suggestions [29] and believed to be practically possible within the limits of the project. Further, it should enable us to assess rates of recruitment and retention to a reasonable degree of precision; assuming an attrition rate of around 20%, a sample of 60 would provide an estimate with 95% confidence interval of width 20% (i.e. +/− 10%). For a recruitment rate of around 50%, the interval width would be around 25% (i.e. +/− 12.5%).

### Randomisation

Randomisation will be coordinated remotely by the Norwich Clinical Trials Unit (CTU). Participants will be randomised in a 1:1 allocation ratio, using a stochastic minimisation algorithm to minimise imbalance between groups in baseline symptom severity, gender and study site. Allocation will be managed by the Data Management Team at Norwich CTU via a web-based system; it will not be accessible by anyone outside of this team, including the research team, trial therapists and participants; thus, allocation concealment will be maintained.

### Blinding

Research practitioners collecting follow-up data will be blind to the participant’s treatment allocation. Another member of the research team will pass details of allocation to the clinical service. Given the nature of the intervention, it will not be possible for participants and those involved in delivering the intervention to remain blind. Following allocation, all participants in the study and therapists will be asked not to reveal the group to which the participants were randomised to the research practitioner. Participants will be reminded at the beginning of each contact with the research practitioner post-randomisation not to disclose their allocation. Any potentially unblinding data will be stored separately in a database to which the research practitioner will not have access. In the case of accidental unblinding during the research process, a second research practitioner will complete the outstanding measures.

### Data collection

#### Outcome measures

Participants will be assessed at baseline (pre-randomisation) and followed up at 10 weeks and 23 weeks, with an additional 5-week follow-up (online with telephone support). The following outcome measures will be used: demographic characteristics of young person; Kiddie Schedule for Affective Disorders and Schizophrenia (K-SADS) depression section to assess for presence of DSM depressive disorders [30, 31]; Revised Children’s Anxiety and Depression Scale (RCADS) to assess self-rated severity of symptoms across depression, anxiety disorders and OCD [32]; Family Assessment Device (FAD) to measure quality of family relationships of participants [33]; Cambridge Friendships Questionnaire (CFQ), which measures quality of peer relationships of participants [34]; Employment, Education or Training in previous 4 weeks (NEET status), to capture levels of inactivity amongst young people who are not in work, education or training; Short Warwick-Edinburgh Mental Wellbeing Scale, which will measure mental wellbeing [29]; Modified Client Service Receipt Inventory (Modified CSRI), which will record information on service utilisation, income, accommodation and other cost-related variables [35]; and Health-related quality of life measured using the Child Health Utility 9D (CHU9D) [36].

The RCADS [32], FAD [33] and CFQ [34] will be repeated at an online week 5-follow-up. All outcome measures will be repeated at week 10 and 23 follow-up except for demographic characteristics of young person and the K-SADS depression section. Information about gender of therapist, attendance/non-attendance at planned therapy sessions and location of sessions will be collected by therapists in both treatment arms.

Table 1 shows the schedule of enrolment, interventions and assessments in accordance with the SPIRIT guidelines.

**Table 1:**
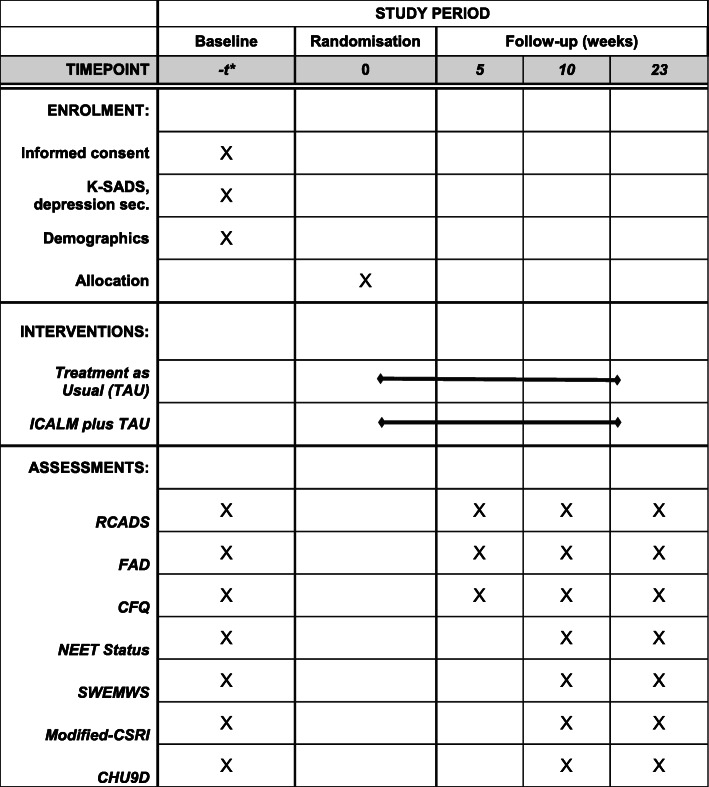
SPIRIT Schedule of enrolment, interventions, and assessments

*The duration between initial screening and randomisation will vary depending on the time it takes a participant to complete baseline measures. However, we anticipate that all participants will be randomised within 4 weeks of their initial screening appointment

### Health economic data

We shall measure use of NHS and community resources related to mental health. All resources required to implement the intervention, including providing training, ongoing clinical supervision, staff time to deliver the intervention, any consumables and materials required and any other necessary expenditure will be captured. We will use a modified version of the Client Service Receipt Inventory (CSRI) [35] completed at each follow-up time point (baseline, 10 and 23 weeks). We shall use the CHU-9D [36] to measure health-related quality of life (HRQoL), which will be administered at baseline, week 10 and week 23.

### Process evaluation data

The trial will employ a mixed-methods ethnographic process evaluation to (a) provide a description of how IPC-A and TAU are delivered, (b) assess implementation and theoretical fidelity to the IPC-A model over time, (c) observe how delivery is shaped by the context of differing service models, (d) identify any harms arising from treatment (including end of treatment) and (e) establish the extent and source of any contamination of the control arm.

Data collection methods will include:

- Site profile questionnaires (one per provider administered at the beginning and end of the trial)
- Observations of IPC-A training workshops and supervision
- Video/audio recordings of treatment sessions (both IPC-A and TAU; all treatment sessions will be recorded, subject to consent)
- Interviews with participants (young person and a parent/carer) from the IPC-A and TAU arms (*n* = 20)
- Focus groups with youth mental health workers (one per arm per provider)
- Focus group with wider stakeholders

#### Video/audio recording of treatment sessions

A random selection of therapy sessions (15% in each arm) will be rated by one of the supervisors according to the IPC-A Audiorecording Rating Scale [27], to monitor implementation fidelity to the IPC-A treatment model and to assess the degree of contamination. This scale includes ratings of techniques for the assessment (e.g. ‘Complete an Interpersonal Inventory’) and ending sessions, for specific focus areas (e.g. ‘Exploration and discussion of differences in expectations’ for relationship disputes), and general IPC techniques to be used for all sessions (e.g. ‘Clear focus on depressive symptoms and interpersonal relationships’).

Selection of sessions will be ongoing throughout the study. Feedback will be given to the therapist from the supervisor for IPC-A cases, to aid continued development. This process will be ongoing through the study so such feedback is timely. We accept that therapists in the TAU arm are less likely to submit sessions, but we shall regularly meet with teams and explain the importance of us rating sessions from both arms of the study, and that the aim of this is to check what TAU is and whether it contains IPC-A; the purpose is not to rate the quality of their therapy. Using findings from supervisor’s ratings of IPC-A sessions, a purposive sub-sample of extracts from recordings will also be selected (sampled to include maximum variation in delivery) and qualitatively analysed to evaluate theoretical fidelity.

#### Young person/parent interviews

Twenty young people participating in the RCT (10 per arm) will be invited to take part in in-depth interviews after their final assessments (23-week assessment). A separate interview will be undertaken with a parent/carer (all parents whose child participates will be invited). These parents/carers will complete a separate consent form for this interview. The research practitioner will have ongoing dialogue with parents/carers about their potential participation in the interviews during the follow-up visits. Participation in the interviews will be ascertained at the final research assessment. Participants will be asked about their experience and views of the process of accessing help, the content of sessions, contacts had in addition to study therapy sessions, how they feel they have benefitted or not from receiving the intervention, the experience of ending therapy and suggestions for improvement.

As with all clinical trials, there remains the possibility for some participants to withdraw from the study. There is a process in place to accurately record participant withdrawals/dropouts from the study. It will be established whether participants withdraw from the therapy (either IPC-A or TAU) and/or from the whole trial. Those who withdraw from therapy only will be followed up and invited to participate in the process evaluation interviews, while those who withdraw from the whole trial will be lost to follow-up and interviews.

#### Staff focus groups

Following completion of delivery of the IPC-A and TAU arms, focus groups will take place in participating services to understand staff perspectives of each study arm. These will be separate for IPC and TAU therapists. For IPC-A therapists, discussion will focus on barriers and facilitators to successful delivery, experiences and views of intervention sessions, additional work required to support delivery of IPC and suggestions for improvement. For TAU therapists, discussion will focus on how TAU is delivered, the additional support YP in TAU have received and their awareness and perspectives of IPC-A.

#### Focus group with professional stakeholders

At the end of the study, an additional focus group will be conducted with commissioners, education representatives and service managers to review study findings and discuss implementation barriers and sustainability of implementation.

#### Site profile questionnaires

Questionnaires at the beginning and end of study will aim to understand the broader service context in which the intervention is delivered, including TAU for young people with mental health needs; policies, protocols and procedures used by staff; numbers of YP with mental health needs and proportion with depression; training and experience of staff in treating depression in YP; and allocation and distribution of staff to support YP with mental health needs.

### Analysis

#### Statistical

Recruitment and retention rates will be estimated with 95% confidence intervals (CIs). Assuming sufficient information, time until drop-out will be analysed using ‘time-to-event’ methods, i.e. in an effort to identify baseline factors likely to be related to drop-out. The proposed primary outcome measure for the definitive RCT is the RCADS depression score at 10 weeks. Although the proposed study is not designed to assess efficacy, the mean between-group difference will be estimated using a general linear model including baseline RCADS depression score and treating therapist as a random effect. A 95% CI will be constructed to assess whether the treatment benefit is feasibly greater than the minimal clinically significant difference, i.e. whether or not it is included within the CI. A similar approach will be undertaken for the secondary outcome measures. The rate of completion of each outcome measure will be reported. If appropriate, depending on the proportion of missing values, multiple imputation will be undertaken and between-group differences re-estimated as a sensitivity analysis. Further parameters, such as within group variation, needed for the design of a subsequent full-scale trial, will also be estimated. All statistical analysis will be undertaken using STATA.

#### Health economic analysis

As this is a feasibility study, it will not be possible to demonstrate the cost-effectiveness of the intervention because the study will not be powered to demonstrate effectiveness. However, we shall collect information to inform the design of the economic evaluation planned for the future definitive trial. This will yield useful information, such as the likely cost of the intervention and key components of resource use. It will also inform the design of health economic data collection instruments in the future fully powered trial.

The resources required to provide the interventions (IPC-A and TAU) will be recorded. These would include training, ongoing clinical supervision, staff time and salary costs required to provide the intervention, consumables and materials required, and any other necessary expenditure. Each session offered (and its location) in both arms will be explicitly recorded. Recording of all events will be built into the design of the study and study CRF. These will be combined with appropriate unit cost data to provide an estimate of the cost of providing IPC-A. It will also be possible to conduct scenario analyses to estimate changes in the cost of provision if any assumptions about how the service is provided are changed. It will be important to measure any resources related to participants’ mental health in both the intervention and control groups. This will be conducted by means of a modified CSRI [35] conducted at baseline, 10 and 23 weeks. The time frame requested for the baseline and 10-week CSRI will be any use of services in the last 10 weeks. For the 23-week assessment, the time frame will be the last 13 weeks. To reduce burden on participants, the a priori aim is to make the modified CSRI as simple as possible but to still capture relevant and important service use. Any modifications made will be made in consultation with other ICALM investigators. The CSRI will be collected by means of a face-to-face interview.

Resource use data will be analysed to highlight any potential areas of differences between trial arms in use of NHS and social care services, including emergency department attendances. The measure of health-related quality of life (HRQoL) used in this study will be the CHU-9D. One important outcome of the feasibility study will be an assessment of the suitability of this instrument and the modified CSRI for use in a future full-scale trial.

#### Process evaluation analysis

A linguistic ethnographic methodology [37, 38] will be employed to analyse how relationships, roles and moments of intervention delivery are organised within the contexts of delivery. This will be achieved by (1) setting out macro, meso and micro contextual features relevant to implementation within each provider; (2) targeting where likely tensions in implementation are likely to occur at each contextual level; then (3) searching for ‘disruptions’ to targeted activities involved in intervention delivery; and (4) considering the consequences of these disruptions for how the intervention was implemented and the implications of these for scaled up implementation in a future definitive trial.

The linguistic ethnographic process evaluation methodology combines strengths of linguistics and ethnography to systematically investigate human behaviour within context. A particular strength is that it provides methodological tools for empirically exposing relationships between talk, non-verbal behaviour and the contexts in which such behaviour is produced. This is particularly helpful for evaluating the interpersonal counselling intervention, which trains local authority and other non-NHS services to communicate effectively with adolescents.

To manage the quality and range of data collected as part of the process evaluation, analysis will involve working laterally across data types. We will seek to provide a broad description of intervention delivery but, instead of allocating equal time to the analysis of each case, we will focus on identifying ‘telling cases’, triangulating and looking for connections between data. The analysis of qualitative data will be iterative, moving between data collection and data analysis to test emerging theories. Care will be taken to identify and follow up deviant cases which do not fit into emerging theories. Emerging theories and the relationship of the data to the conceptual literature underpinning the intervention will be discussed and refined at team meetings throughout the research.

Researchers’ field notes from observations of training and supervision of IPC-A therapists will be analysed thematically to provide a detailed description of process and content of staff training and supervision. Interviews with individual young persons and focus groups with staff and stakeholders will be transcribed verbatim and thematically analysed with the aid of NVivo software. For intervention arm participants, we will then develop a coding scheme to evaluate how the process and content of IPC-A as delivered by the youth mental health workers have functioned from the participants’ perspective. In the control arm, we will assess how participants experienced the treatment as usual provided by their youth mental health worker and any other sources of support used. A constant comparison approach will be adopted, working iteratively between data obtained from different interviewees within and between implementation sites.

The ratings of IPC-A sessions will be used to monitor implementation fidelity to the IPC-A treatment model and to assess the degree of contamination. If contamination of the TAU arm is identified, data generated through observations, interviews and focus groups will be used to explore the mechanisms by which contamination occurs and how this might be mitigated against in a future trial. To evaluate theoretical fidelity, the purposive sample of extracts of recorded IPC-A sessions will be transcribed according to Jeffersonian conventions and subject to conversation analysis in order to identify how IPC-A components are communicated by therapists and received by young people, including how the mechanisms of the IPC-A intervention function to affect change within and across individual counselling sessions.

By framing the analysis of intervention implementation within a macro, meso and micro contextual framework, we will be able to make the transition from the identification of routines and patterns of use in the specific services participating in the current study to theoretical explanations of how different structural relations and mechanisms of the intervention organise moments of delivery, which then impact on specific outcomes. In drawing case comparisons across participating services, we will develop hypotheses about why the intervention is linked to outcomes which we can test in a future definitive trial. This may lead us to identify factors which are plausibly and/or consistently related to successful or unsuccessful delivery of the components of the intervention. Emerging theories and the relationship of the data to the theory underpinning IPC-A will be discussed and refined in team meetings throughout the research.

### Patient and public involvement

Protocol development was informed by two PPI events attended by 14 young people, most with personal experience of accessing mental health services. The first event was held at a local school and the second with members of Suffolk Children & Young People, Action and Transformation (CAT) group. The young people we consulted stressed the inadequacy of current mental health provision for young people and supported the idea of extending access to treatment by training existing staff working with young people to deliver IPC-A. They told us that knowing workers have appropriate training is important to building trust and that they would prefer to be treated somewhere familiar to them rather than attend a specialist clinic.

We have engaged two Youth Advisory Groups, made up of young people with personal experience of low mood, for the feasibility RCT stage. Based in the two counties in the UK where the study will be conducted, each group is made up of 4–5 members, and they have been involved and will continue to be involved in key decisions regarding the conduct of the trial, interpretation of the results and dissemination of the findings. The Youth Advisory Group will be facilitated by ST who will be the dedicated PPI lead co-applicant for the trial. ST is a Co-Production Advisor who works as part of Suffolk County Council’s Engagement Hub. She is skilled in facilitating the engagement of young people with mental health needs. ST will act as a point of contact for the young people involved and ensure their welfare by offering emotional support and signposting to appropriate services if young people need further support as a result of the sensitive nature of the research.

Two representatives of the Youth Advisory Group sit on the trial steering committee (TSC). They will be supported by ST to prepare for and attend these meetings. Involving this number of young people will increase the breadth of experience and skills and allow for group members to support and encourage each other, while ensuring that all members are able to contribute meaningfully; it will also allow for attrition, as young people choose to leave the group.

Based on our experience in previous trials, we anticipate that involving young people with relevant lived-experiences as members of the research team will enhance our ability to successfully recruit and retain participants and to effectively communicate the study’s findings to a broad range of stakeholders. The Youth Advisory Panel will be involved in hosting the public dissemination event and in preparing reports of the findings for trial participants and the public.

### Progression criteria

#### Feasibility outcomes

The primary output of this feasibility trial will be the design of the subsequent full-scale trial. The TSC will assess the trial against the following criteria and make recommendations regarding the suitability of the proposed design for the full-scale trial, based on the extent to which these criteria are met.

- Recruitment rate is at least 80% of target
- At least 70% of those randomised to receive the intervention attend at least three therapy sessions within the 10-week treatment window
- Follow-up assessments are completed by at least 80% of participants at 10 weeks and 70% of participants at 23 weeks
- At least 80% of IPC treatment sessions reviewed meet treatment fidelity criteria
- Contamination of the control arm can be sufficiently limited for individual randomisation to be justified
- The mean RCADS depression scores of the IPC-A and TAU groups at 10 weeks are indicative of a clinically significant difference in depression (3 points)

The rate of completion of each outcome measure will be calculated and acceptability assessed via the process evaluation. We shall estimate the expected cost of the intervention and likely drivers of cost. The results of the feasibility study, the views of participants in the stakeholder focus group and guidelines from the Medical Research Council (MRC) on developing and evaluating complex interventions, will inform research design for subsequent research. If the proposed method is unable to limit contamination adequately, then a cluster trial or stepped wedge design will be considered. We will recommend an internal pilot study if outcomes from feasibility suggest that substantial changes to the protocol are required before progression. If the above criteria are met, we will apply for funding to progress to a multi-site, assessor-blind, RCT of the effectiveness and cost-effectiveness of IPC-A in comparison to TAU for adolescents presenting to non-specialist services with depressive symptoms, informed by our feasibility results.

Ultimately, if a future trial demonstrates IPC to be effective, an IPC-A training programme could be implemented nationally. This would facilitate rapid expansion of the therapy workforce, increasing access to evidence-based treatment for adolescent depression.

#### Ethics and dissemination

We obtained ethics approval from the NHS Health Research Authority Cambridge South Research Ethics Committee (UK). The study was reviewed for its scientific design, data collection methods, plan of analysis, risk assessment and management, and ethics. Additional local research approvals were obtained from the two participating County councils in England and all other participating organisations.

Findings of the feasibility trial will be disseminated to trial participants, commissioners, service managers, service users and their parents, clinicians and academics. Dissemination vehicles will include regular study newsletters, a public dissemination event, publications in peer-reviewed journals and presentation at scientific conferences. Study results will also be shared with the National Children and Young People’s Mental Health improvement team.

We shall work with our young advisors to disseminate findings to the public in a way that is accessible to young people, perhaps using YouTube/Instagram/other social media. Young people will be involved in hosting our public dissemination event.

## Discussion

A strong commitment to improve mental health services for children and young people was set out in 2015’s *Future in Mind* [39] and 2016’s *Five Year Forward View for Mental Health* [40], and reaffirmed in the recent Green Paper, *Transforming children and young people’s mental health provision* [41]. The *Five Year Forward View* set the ambition to expand the provision of community-based psychotherapy to an additional 70,000 young people by 2020/21, necessitating a substantial expansion of the therapy workforce. *The NHS Long Term Plan* [42] further continues to invest in the improvement and development of CYP MH services. Achieving this goal of improved access to treatment will require a joint-agency approach and a greater focus on providing evidence-based interventions outside of specialist CAMHS.

Utilising the skills of the existing staff members by training them to deliver evidence-based interventions will be essential to meeting workforce requirements. However, there is currently no evidence to support decisions about which interventions these staff members should be trained to deliver. The proposed research aims to contribute to this evidence-base. The study is in line with the Department of Health’s *Framework for Mental Health Research*, which recommends that research should focus on early intervention and the voluntary sector. We propose that IPC-A could be an effective treatment for young people with depression, which could be delivered by such non-specialist services.

In order to contribute to the evidence-base for interventions for adolescent depression that can be delivered outside of specialist CAMHS, an evaluation of the effectiveness and cost-effectiveness of IPC-A is needed. However, before a definitive randomised controlled trial can be justified, there are a number of feasibility questions that need answering. In particular, the process of recruiting, randomising and conducting research assessments is not part of normal practice in non-specialist services, and it is important to demonstrate that this is feasible.

## Data Availability

Not applicable.
